# Vulnerability and risk: reflections on the COVID-19 pandemic

**DOI:** 10.1590/S1980-220X2020045203777

**Published:** 2021-07-26

**Authors:** Clara Juárez-Ramírez, Florence L. Théodore, Héctor Gómez-Dantés

**Affiliations:** 1 Instituto Nacional de Salud Pública de México, Centro de Investigación en Sistemas de Salud, Cuernavaca, Mexico.; 2 Instituto Nacional de Salud Pública de México, Centro de Investigación en Nutrición y Salud, Cuernavaca, Mexico.

**Keywords:** Risk Taking, Health Vulnerability, Communicable Disease Control, COVID-19, Assunción de Riesgos, Vulnerabilidad en Salud, Control de Enfermedades Transmisibles, COVID-19, Assunção de Riscos, Vulnerabilidade em Saúde, Controle de Doenças Transmissíveis, COVID-19

## Abstract

At the end of December 2019, SARS-COV-2 virus was identified as responsible for the COVID-19 pandemic. The rapid spread of transmission exposed structural failures of modern societies and of the health systems in preventing and containing a health threat. Scientific discussion has focused on the search for a vaccine, but less on understanding the social response to the current global threat and fear of outbreaks. In this essay, we reflect, based on the social sciences, on the importance of linking three concepts: vulnerability-perception-risk. This is necessary to develop preventive strategies appropriate to population circumstances, especially with the most vulnerable population, in favor of health equity.

## RESUMEN

A finales de diciembre 2019 se identificó el virus SARS-COV-2 como responsable de la pandemia de Covid-19. La rápida expansión de la transmisión puso al descubierto fallas estructurales de las sociedades modernas y de los sistemas de salud para prevenir y contener una amenaza sanitaria. La discusión científica se ha concentrado en la búsqueda de una vacuna, pero menos en comprender la respuesta social ante la amenaza globalizada actual y el temor a los rebrotes. En este ensayo reflexionamos, desde las ciencias sociales, sobre la importancia de vincular tres conceptos: vulnerabilidad-percepción-riesgo. Esto es necesario para desarrollar estrategias preventivas adecuadas a las circunstancias poblacionales, especialmente con la población más vulnerable, a favor de la equidad en salud.

## RESUMO

No final de dezembro de 2019, o vírus SARS-COV-2 foi identificado como responsável pela pandemia de Covid-19. A rápida propagação da transmissão expôs falhas estruturais das sociedades modernas e dos sistemas de saúde na prevenção e contenção de uma ameaça sanitária. A discussão científica tem se concentrado na busca por uma vacina, mas menos na compreensão da resposta social à atual ameaça global e ao medo de novos surtos. Neste ensaio refletimos, a partir das ciências sociais, sobre a importância de associar três conceitos: vulnerabilidade-percepção-risco. Isso é necessário para desenvolver estratégias preventivas adequadas às circunstâncias da população, principalmente junto à população mais vulnerável, em prol da equidade na saúde.

## INTRODUCTION

At the end of December 2019^(1)^ in Wuhan, China, SARS-COV-2 virus was identified as responsible for the COVID-19 pandemic. As the pandemic spread, containment, mitigation, and control strategies were established as the epidemic spread in each country and with various modalities that included from border closures to mandatory confinement. Measures intensity to prevent contagion was established based on the estimates of the potential growth of transmission, obtained from mathematical models rather than from a clear understanding of the determinants of social dynamics and the preventive behavior to mitigate the virus transmission progression. Academic discussion and scientific research have focused on the search for a vaccine or a treatment to manage the medical emergency, without reflecting on the pressing need to communicate the risk to the population and its implications to the response from the health care systems.

However, contemporary authors highlight the impact on different spheres of society and the intensification of the civilizational crisis, which in the pandemic is expressed by increasing inequalities among people, manifest among those who were able to follow the recommendations to avoid contagion (such as stay at home), and those called “essential workers” (public cleaning services, home delivery workers, market workers), but who are paradoxically the most vulnerable^(2–3)^.

The rapid spread of transmission exposed the structural failures of modern societies and of health systems regarding their ability to prevent a health threat and their power to contain it. Social pressure has even questioned the leadership of scientific organizations and international bodies, such as the World Health Organization. In the region of the Americas, infections continue increasing, namely, in decreasing order: United States (23,556,676); Brazil (8,488,099); Colombia (1,908,413); Argentina (1,799,243) Mexico (1,641,428); Peru (1,064,909) according to figures from the World Health Organization updated as of January 20, 2021^(4)^.

Within this context, the present reflection was triggered by the following question: How are we going to organize ourselves to promote the adoption of new cultural standards and social behavior that impact on the risk of contagion?

In this regard, there are at least two interconnected and essential approaches: one is the establishment of preventive measures from the governing level of health care, the other is the adoption of health measures and their individual practice but for the collective benefit. To implement the adoption of individual risk prevention measures, it is necessary to understand the elements involved in the *risk perception* of virus contagion, differentiated depending on the place people occupy in society.

The objective of this essay is to reflect on the process by which individual and collective responses are generated to the risk of contagion of COVID-19, thus producing basis for the design of possible communication strategies.

The arguments are presented in the context of the last three pandemics and the epidemiological and social responses that they aroused, risk perception seen as a social and cultural phenomenon, and the vulnerability as a determinant of the risk of contagion.

### a) Three pandemics, epidemiological characteristics and social responses

Charts 1 and 2 present the epidemiological characteristics and the social construction of meaning and response to three recent pandemics.

On the basis of the comparison of the epidemiological and social responses to the HIV/AIDS and H1N1 pandemics, as shown in Chart 1, we can analyze that biological and social discourses intersected each other when faced with the interpretation of a health problem in an era where the world organized itself immunologically, marking limits, crossings, thresholds (economic and social), and erecting walls against migration or trade, which allowed rejecting or expelling the unwanted, the foreigner, or the hazardous^(8)^.

If, in the past, societies considered themselves impermeable and protected, currently they are vulnerable, fragile, and with no control measures at their disposal to understand, predict, let alone domesticate the dangers or threats generated in other places but that move, are exported, travel, and invade with no great geographic, populational, social, or cultural resistance. In a globalized world, it is impossible to ensure safety of one or many countries, much less regardless of what happens in the rest of the world^(11)^. Reopening generates risks, fears, and turns the options of progress into unforeseen risks. As can be seen in Chart 2, the different discourses built from stigmatization, due to the lack of adequate information in the initial stages, generated opinions among the population that were not favorable for the implementation of risk prevention strategies, which shall be a lesson for future pandemics.

### b) Risk perception seen as a social and cultural phenomenon

Based on Phenomenology, by Merleau-Ponty, perception is a process by which the body biology allows the apprehension of the external world through the senses and the acquisition of language, which allows human interaction^(12)^ to communicate subjectivity^(13)^. It is thought that people create and recreate it on a daily basis through their practices and according to their individual motivations and intentions^(14)^.

#### Danger-risk:

To refer to danger/risk, we have chosen some sociologists who address the subject, seen in Chart 3, who agree on defining “modernity” as the current stage of the development of societies: characterized by individualization and the loss of collective meaning. Zygmunt Baumann and Ulrich Beck suggested that societies advanced towards greater provision of “life options”, but - paradoxically - greater individualization is characterized by more “uncertainty”, and is the distinctive feature of modern society^(15–16)^.

On the basis of this sociological perspective, Luhmann addresses *danger* as societies managed to contain the environmental vicissitudes (natural disasters) and epidemic diseases (plague, smallpox). The “risk”, on the other hand, is a notion - constructed by the development of science and technology - that makes us feel or think that these events can be controlled, and they stop representing a danger because it is possible to influence them to avoid damage. In this regard, danger establishes itself, it is not chosen^(15)^, and therefore it generates fear^(16)^ as a natural reaction expressed emotionally, related to cognition and to the sociocultural context^(17)^. In the current societies, *risk* according to Le Breton, beyond the possible damage generated by living with it on a daily basis, unpredictably involves decision-making based on information; it is linked to a personal initiative of rationality that implies relying on “evidence”, therefore it should not be classified as an “irrational” response or linked to people’s reckless behaviors, since all responses have their own logic and meanings.

The individual response to what we perceive as a danger or risk takes place through our social learning, about what is defined socioculturally as such. In this respect, “risk perception” is a cognitive category of a biological type, but the process by which one names it and acts on what is perceived has a social origin and is built on the basis of standards, values, emotions; learned in the context where one is born, grows up, and dies.

**Figure 1 -:**
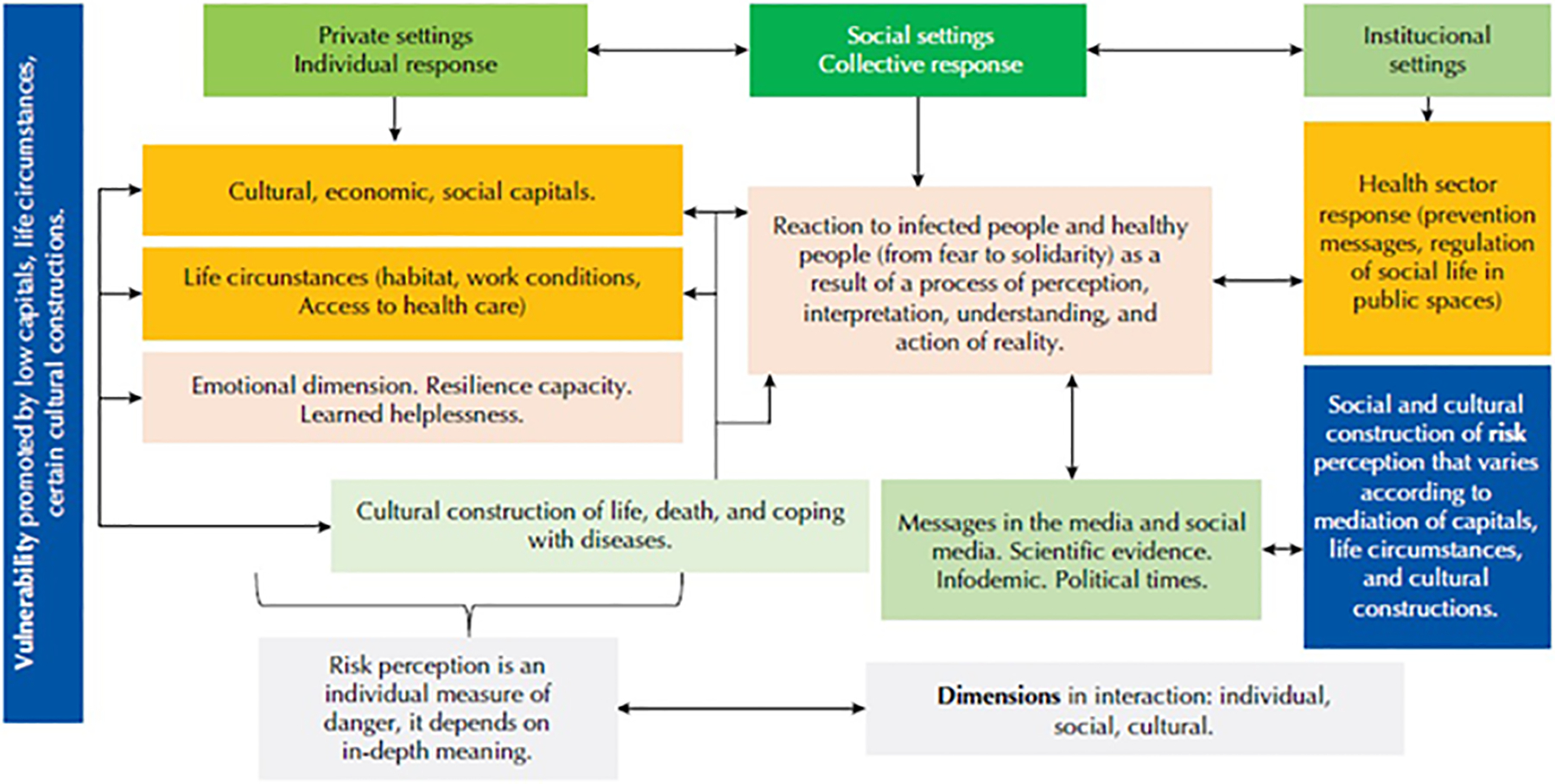
Risk perception and the mechanics of the individual and collective response to COVID-19 according to the degree of vulnerability.

Individual reactions in response to the threat of contagion depend to a great extent on the *capitals* (economic, social, cultural) (Figure 1); Pierre Bourdieu used the category *habitus* (dispositions, schemes for acting, thinking, feeling) to explain that the position of people in social space was defined by the type and volume of these capitals^(18)^. Thus, the availability of information, the level of knowledge that one has about the pandemic and how much *meaning* the information available brings us about its possible immediate repercussions influence the individual response on whether to adopt the health measures.

In addition to what has been said, Mary Douglas proposes that *risk* is constructed through a process of *perception*, interpretation, understanding, and action based on individual experience from which people elaborate their system of practices regarding how to take care of themselves and their family^(19)^. The more familiar and “domesticated” the environment becomes, the belief in a ‘subjective immunity” is established; that is, as people become more familiar with the context, the possibility of the threat is minimized. This takes place as a survival strategy for people, to continue carrying out their daily activities and continue living within “normality”; otherwise, we could not accept the uncertainty of what we perceive as a threat in everyday life, as the consequence would be paralysis of every activity^(19–21)^ which, in the case of the COVID-19 pandemic, is not a possibility for everyone.

According to the authors referred to in Chart 3, we can summarize the following: 1) all groups develop preventive practices (not necessarily aligned with sanitary recommendations) in the event of an identified danger; 2) risk perception is based on a sensory register and at the same time denotes culture^(23)^, which implies a process based on perception, interpretation, understanding, and actions involving elements of different scales, from the most micro with processes of interpretation varying according to capitals to the macro with the political and media contexts.

### c) Vulnerability as a determinant of contagion risk

“Vulnerability”, understood here as the layers of risks that accumulate, represents a real risk based on the differential access that one has to resources to protect health, but it is also a sociocultural condition that builds *meaning*, from where to look at the world, perceive the risk of contagion, and act accordingly; it is not only a condition limiting people’s autonomy and decision-making, the participation of the State and its leading role in the organization of the health system shall also be included in the analysis. In this regard “risk”^(24)^ indicates the probabilities of contagion due to exposure, from an epidemiological perspective; on the other hand, “vulnerability” is a concept that implies susceptibility to illness but as a consequence of sociocultural frameworks that allow it, and the limitation of resources to face it; based on this perspective, vulnerability implies social inequity.

Other authors identify the incorporation of the concept of “vulnerability”^(25)^ into the field of public health based on the HIV-AIDS epidemic in the 1990s, due to the need to design comprehensive interventions that included social movements in defense of human rights^(26)^. Subsequently, systematic reviews locate the use of the concept for the analysis of chronic non-communicable diseases; in both cases, the concept was used seeking a more comprehensive perspective of the patients’ reality. This way, COVID-19 pandemic evidenced the inequalities and inequities in health care present in different countries, as well as the importance of recognizing the differentiated effects according to sex, economic, social and ethnic conditions. It began to spread throughout the world through people with more economic resources, who can travel, have a vacation, or go on business to other countries; however, it affected each country in different ways, depending on the opportunity and intensity of the measures of mitigation and containment established. In the poorest countries, with greater difficulties in the organization and infrastructure of health services, there were greater problems when facing the pandemic. In contrast, in the high-income United States, the highest mortality was among those of ethnic origin such as the Navajo nation, Afro-descendants, and Latinos^(27)^.

Regarding preventive measures against the risk of contagion of COVID-19, not all people have been able to follow the health recommendations. In Latin American countries, as in the rest of the world, social vulnerability, marginalization due to ethnicity, and poverty environments were also evidenced. Another vital situation that generates risk of contagion is the work environment. In this group there are wage earners with social security but also street traders. On the other hand, we have the indigenous population and, among them, various levels of vulnerability. There are those who live in more urbanized towns, but also those who live in the mountains, in the jungle, in rural areas.

In this respect, the differentiated impact of COVID-19, based on having more or fewer resources to deal with it, also allows to problematize the link between how risk is perceived and social vulnerability, that is, How is perception of the risk of COVID-19 constructed when on a situation of vulnerability and vice versa? What actions can people in conditions of greater vulnerability take to protect themselves from the risks of becoming ill? What are the limits of these actions, despite the will to do them, when going through economic subsistence needs?

Thus, from a social justice perspective, vulnerability “is the degree to which the distinct social classes are differentially at risk”^(28)^. It is the result of interactive and dialectical processes between people and their social context, marked by limitations in access to different areas of social protection such as health and education, which builds particular ways of seeing and acting in the world.

In the field of bioethics, the ‘vulnerability’ of certain population groups makes them more susceptible to any form of damage^(29)^, being able to accumulate different types of vulnerabilities^(30)^. This way of visualizing risks, based on these layers of vulnerability, allows us to have a panoramic perspective to act with preventive measures. As a summary, and based on everything presented throughout the essay, we present the perception of risk and the mechanics of the individual and collective response to COVID-19 according to the degree of vulnerability in Figure 1, in which we want to emphasize the importance of reflecting on the implications that the search for short-term/technical solutions may have on the health systems organization, specifically (public/political scenario) and regarding the expected response from society (social scenario); especially when solutions will take many more months to become a reality for everyone (private/individual scenario). They are interconnected and essential areas to address: the establishment of preventive measures from the governing level of health administration (collective context, institutional settings), that related to the individual adoption of these measures (new behaviors) and the importance of practicing them for the collective benefit.

Given the heterogeneity of vulnerabilities, a challenge for the health system is to organize the health response in the preventive field tailored to the different “vulnerable” groups; but understanding that although “perceiving a risk” is a cognitive category of a biological type, the process through which that “risk” is designated and acted upon as perceived has a social origin, built from standards, values, emotions, learned in the context where one is born, grows up, and dies.

Starting from a comprehensive perspective of the situation of social vulnerability in which the different population groups are found, it is necessary to know how they construct the perception of risk, as an essential notion to develop effective communication strategies to promote the adoption of measures that prevent the contagion. A critical strategy shall involve several dimensions of social life: the individual-community sphere; the organization of the health system, both within (infrastructure, human resources) and outside (risk communication, education, and health promotion).

With the COVID-19 pandemic, risk communication shall be extended to the re-organization of spaces in all collective areas (hospital, “essential and non-essential” work, religious, sports, among other areas), to keep the social distancing measures and reduce exposure to contagion (Figure 1). In these prevention tasks, the role of nursing is essential.

## CONCLUSION

This essay aimed at discussing that risk perception and exposure to it are differentiated according to the conditions of vulnerability experienced; in addition, health actions/behaviors that people perform individually are in line with the sense and meaning that the disease has for them. It is evident that to face the pandemic a conscious exercise of social relearning is required in different areas. As shown in this essay, based on the comparison of the three pandemics, it can be said that risk perceptions of contagion change as the knowledge about the disease advances. However, to follow the promotion of prevention measures, it is important to understand the sociocultural dimension of risk perception and its close link with the different vulnerabilities. In addition, to adopt the recent proposals on the need for public health to identify how intersubjectivity influences the behavior of peers. This is necessary to carry out a preventive approach that is more focused on the actions that are generated from personal needs and not based on homogeneous health responses, as evidenced by the HIV-AIDS epidemic^(31)^. Additionally, to begin to identify the lessons learned from the COVID-19 pandemic and those that are likely to come, it is important to reflect on the urgent need to strengthen health systems aiming at health equity. We hope that this document contributes to the regional discussion that has taken place in the field of public health and community health.

## Figures and Tables

**Chart 1 -:** Epidemiological characteristics of the three recent pandemics.

Notable epidemiological factors	HIV/AIDS (1980’s)^(5–6)^	Influenza (H1N1– 2009)^(7)^	COVID-19 (2020)
**Route of transmission**	Sexual, infected blood, breast milk.	Saliva droplets from sick people.	Saliva droplets, exhalation of particles from asymptomatic and sick people.
**Lethality level**	High (before the advent of antiretroviral treatments). Since the beginning of the pandemic and until 2019, 32.7 million deaths are estimated in the world according to UNAIDS.	Low/moderate in all age groups. Until 2018, 75,000 deaths were calculated.	High in people over 60 years of age and with comorbidities such as diabetes and hypertension. As of December 23, 2020, there were 76,382,044 cases and 1,702,128 deaths caused by the disease in the world (WHO)^(4)^.
			Low in children
**Main symptoms**	From asymptomatic to severe immunodeficiency.	Fever, sore throat, nasal congestion, cough, muscle aches, headache, chills, fatigue.	Nasal secretions, fever, tiredness, smell and taste loss, difficulty breathing, among others.
			Asymptomatic forms.
**Treatment**	High cost, very toxic initial therapy of limited efficacy (AZT); at the end of the 1990s, more effective drugs were combined that were widely distributed free of charge. It became a treatable chronic condition.	Tamiflu (oseltamivir) available since the first cases.	There is no specific treatment. Antivirals under evaluation Intensive care, management with mechanical respirators.
**Prevention and behaviors promoted with the population**	Vaccine: does not exist	Vaccine: Rapid Development October 2009.	Multiple vaccines in development. Technological innovations.
	Use of condoms during sexual intercourse.	Sneeze etiquette.	Sick people isolation.
	Safe blood for transfusions and banning of blood trade.	Isolation of patients with symptoms of respiratory tract conditions.	Sneeze etiquette, hand washing, mask.
	Use of disposable syringes.	Use of protective barriers: gloves, face masks.	Voluntary confinement. Restriction of social life and use of public spaces.
	As of 2010, according to UNAIDS recommendations, “combined prevention” in HIV/AIDS of biomedical, behavioral, and structural interventions.	Avoid physical contact: greetings, hugs, kisses.	Physical distancing: greetings, hugs, kisses.
	-	-	Keep the house ventilated and clean.
	-	-	Disinfect commonly used utensils and surfaces.
**Health measures**	Banning of blood trade.	Unique health contingency.	School closings.
	Free diagnostic tests.	School closings.	Social distancing campaign.
	Creation of special centers to attend cases on an outpatient and inpatient basis.	Suspension of massive activities (religious events, movie theater, theater, sports events).	Suspension of “non-essential” activities
		Limited duration of measures	Quarantine
	-	-	Creation of traffic light model to identify areas of higher risk.
	-	-	Community approach, from the Primary Care.
**Institutional interventions**	Training, diagnosis, treatment of health personnel at all levels.	Adequacy of clinical facilities without worrying about hospital burden.	Hospital transformation.
	AIDS Clinics.		Private hospitalization and care services.
	Educational campaigns.	Accessible vaccine and treatment.	Acquisition of specialized equipment.
	Provision of condoms.		Test development: only confirmation, no test strategy, tracing of contacts and isolation of confirmed ones.
**Participation of international organizations and society**	WHO; UNAIDS; Civil society with NGOs (Act-Up; Aids) and organized groups to face the epidemic.	World Health Organization (WHO); Centers for Disease Control and Prevention (CDC), United States.	Criticisms to the World Health Organization (WHO).
			Heterogeneous national initiatives against the pandemic.
			Philanthropic influence from international foundations, such as the Bill and Melinda Gates Foundation.

**Chart 2 -:** Social construction of *meaning* and response to pandemics.

Notable social aspects	HIV/AIDS (1980’s)^(5.6)^	Influenza (H1N1– 2009)^(9)^	COVID-19 (2020)
**Risk groups**	People with “risky” sexual practices Stigmatizing burden at the beginning of the pandemic. 4Hs: Hemophiliacs, homosexuals, heroin addicts and Haitians.	Universal susceptibility.	Universal susceptibility.
	People receiving contaminated blood (transfusion, syringes).	Adults older than 65, pregnant women, children, people with asthma.	Comorbidities: hypertension, diabetes, obesity, people with HIV/AIDS, people with asthma, smokers.
	Heterosexual women.	Comorbidity: Heart and cerebrovascular diseases, diabetes, HIV/AIDS, cancer, children with neurological conditions.	Greater biological vulnerability: older adults, pregnant women, people with chronic diseases.
**Population risk perception**	Link and stigma against gay, transgender, sex workers communities, injection drug users, etc.	Disbelief in the face of illness, theory of government conspiracy to generate social control and to obtain economic profit^(7)^.	Heterogeneity: denial of disease, risk factors, and transmission mechanisms; disbelief in protective measures (believed to be exaggerated). Others do understand and protect themselves.
	Safe sex with a condom.		Who are essential (poor and vulnerable), and nonessential (privileged)?
**Related social vulnerability**	People in a situation of social and economic inequity (women, sex workers, migrants).	People in a situation of social inequity who could not suspend their subsistence economic activities.	Health personnel
			People in a situation of social inequity who could not suspend their subsistence economic activities.
		People without access to information.	People without access to health services.
			No schooling.
		People without access to health services.	Afro-descendant, Latin^(3)^ population, native peoples.
			In nursing homes, prisons.
		People without schooling; non-Spanish speakers.	Food processor workers^(3)^.
			Dependent elderly.
**“Sign” of illness**	Kaposi’s sarcoma, extreme thinness	The flu or common cold = influenza.	Invisible, asymptomatic disease, hospital isolation of the sick
**Social reaction**	Fear, stigmatization and rejection of infected people and groups identified as “of risk”.	Fear.	Fear and stigma (Asian population).
		Acceptance of confinement measures.	Irrational rejection of health personnel perceived almost as “vectors”.
		On the international scene, discrimination against Mexicans for Mexico being the epicenter of the pandemic^(9)^.	Uncertainty about the return to “normality” (schools, work, and social life).
		-	Rejection of prolonged confinement (individual right).
		-	Non adherence to prevention measures.
**Popular explanation of disease: search for culprits**	Punishment linked to religiosity towards groups identified as “of risk” (plagued, sinners).	Punishment linked to religiosity towards groups identified as “at risk”^(10)^.	“I don’t see it, it doesn’t exist, I don’t catch it” (COVID-19 youth parties).
		Guilt of those who over-exploit natural resources.	Rich travelers who “imported” the virus from other countries.
		-	Stigmatization of the Asian population (China due to the origin of the virus).
**Risk communication**	Innovative prevention campaigns targeting specific groups (e.g. adolescents, men who have sex with men).	Information by official means.	Daily informative conferences by the government health administration.
			Information platforms on pandemics (cases, deaths, tests, etc.).
			Another level of information comes from the press and social media with false news to generate confusion in public opinion: infodemic, fake news.
	Campaign acceptance.		Political use of the disease. Ideological position on the ineffectiveness of the planning and operation of preventive strategy and care.
			Accountability: questioning of the whole care and prevention process.
			Prevention campaigns aimed at the general population.

**Chart 3 -:** Main contributions of some authors on the *risk* theory.

Author	Main contributions	How does it help to understand the current context?
R. Castel^(22)^	There are two types of risk: *social* (illness, loss of job, death); and *modern* (a product of industrialization and globalization, such as global warming) ones.	Greater individualization of modern societies brings as a consequence more uncertainty about how to face risks.
	Stage of “safe society” through the establishment of the Social Security/Welfare State.	Greater emotional burden on the uncertain future.
	The phase of modernity in today’s societies is characterized by greater “individualization” and loss of the collective sense.	Paradox of the current epidemic: With COVID-19, people are asked to think from the collective (stay home to save the lives of others).
A. Giddens^(20)^	*Modernity is a culture of risk* (p. 35).	Knowledge takes the form of permanent hypotheses about everyday events.
		People build their explanations about COVID-19 based on what they hear in the media.
	Risk and trust, two concepts united in times of uncertainty.	Paradox of the current epidemic: With COVID-19, fake news is mixed with scientific data, infodemic affects people’s trust in the information received to prevent risks.
		Ambivalent.
		Stopping routine and daily activities leads to distress.
		People need to feel confident to perform well.
U. Beck^(16)^	“Global threat situations that arise for all humanity” (e.g. nuclear accidents).	Not only were COVID-19 cases identified in less than three months in most countries, but also their inhabitants were quarantined, at the same time, in their homes.
	“They endanger life on this Earth, and all its forms of manifestation.”	Although the author referred mainly to the danger derived from nuclear activity and the consequences of climate change, COVID-19 has endangered social life, due to the prevention measures that were taken, necessary to reduce the risk of contagion: restricting social encounters, physical contact, etc., which are the essence of human life.
D. Le Breton^(23)^	All societies have developed symbolic systems or a ‘management’ model to eliminate ‘danger’, as in the past: wars, famines, diseases such as the Black Death. To pray (religion), discriminate and blame the other, minimize the threat in relation to other priorities (economy).	Risk control and management has been the object of political struggle between a scientific approach that recommends confinement to ‘flatten the curve’ and to avoid hospitals saturation to be able to attend to the serious forms of COVID-19, and an approach defended by some Heads of State who minimized the danger of the virus and the risk of contagion that this pandemic represents, stating that it was a form of flu, and rejecting quarantine.
	“Risk” is the key element of the modern societies symbolic system and has become the object of political, ethical, and social struggles to define the risky situations and the ways to prevent them.	
Z. Bauman^(15)^	*Intolerance and ambivalence*, two concepts derived from modernity.	Ambivalence results in disarrangement, inner discomfort from not being able to interpret the signs and choose alternatives.
	*Ambivalence:* possibility of relating an event to more than one category.	
	*The disposable lives*.	
M. Douglas^(19)^	*Risk* is built through a process of perception, interpretation, understanding, and actions in the people’s immediate reality.	The population group to whom we want to communicate the health risk shall be defined so as to define strategies.
	Through experience and the family values system, one learns how to take care of him/herself.	As time goes by and we get used to living with the virus and the measures, an idea of ‘subjective immunity’ is created that the measures to prevent contagion can be relaxed.

**Source:** own elaboration from the bibliographic production of the referred authors.
